# Study on Quality Characteristics of *Lonicera* Tender Bud Tea Based on GC-IMS and Electronic Sensory Technology

**DOI:** 10.3390/foods15101686

**Published:** 2026-05-12

**Authors:** Mengxue Li, Li Zhang, Hua Ji, Xue Han

**Affiliations:** 1 Institute of Biotechnology and Food Science, Hebei Academy of Agriculture and Forestry Sciences, Shijiazhuang 050051, China; 13781158059@163.com (M.L.); lizhang@haafs.org (L.Z.); 2 College of Food and Biology, Hebei University of Science and Technology, Shijiazhuang 050018, China

**Keywords:** *Lonicera japonica*, bud tea, active compounds, volatile compounds, flavor quality

## Abstract

As a new type of tea, *Lonicera* tender bud tea currently lacks clear scientific standards for variety selection and harvest time determination, and relevant research on its components is insufficient. This study focused on ‘Beihua No.1’ and ‘Red Honeysuckle’ as research objects and systematically analyzed their quality-related components using GC-IMS and electronic sensory technology. The results showed that: the basic nutritional components of ‘Beihua No.1’ were highest in August, and main pharmacological components peaked in April, with a high content of loganin; the components of ‘Red Honeysuckle’ were optimal in June, with a high concentration of swertiamarin. GC-IMS analysis revealed that ‘Beihua No.1’ contained 71 volatile substances, and ‘Red Honeysuckle’ contained 79 volatile substances, both mainly composed of esters and heterocyclic compounds. Through VIP analysis (VIP > 1), ‘Beihua No.1’ had 18 key differential components, including Methyl non-2-ynoate, 4-methylbenzaldehyde, cyclopentanone, etc.; ‘Red Honeysuckle’ had 28 key differential components, including 2,3,5,6-tetramethylpyrazine, 2-isopropyl-5-methylcyclohexanone, (E,E)-2,4-heptadienal, etc. Cluster analysis confirmed that ‘Beihua No.1’ is suitable for fresh-tasting tea, while ‘Red Honeysuckle’ is suitable for mellow-tasting tea. This study provides scientific support for the high-value development and standardized production of both.

## 1. Introduction

**Figure 1:**
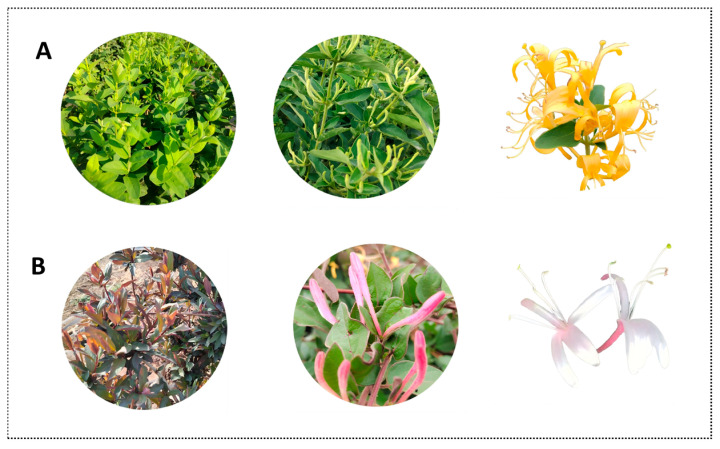
Appearance and flower buds of two *Lonicera japonica* varieties. (**A**) ‘Beihua No. 1’ and (**B**) ‘Red Honeysuckle’.

The dried flower buds or partially opened flowers of most species in the *Lonicera* genus are traditional Chinese medicinal materials, known for properties such as heat-clearing, detoxifying, antibacterial, and antiviral effects. Among them, honeysuckle (*Lonicera japonica* Thunb.) is the most widely cultivated variety in China. In recent years, mutants of *Lonicera* plants have been progressively developed and have gained increasing popularity among consumers [1,2]. ‘Beihua No. 1’ is a new cultivar selected from natural variants of honeysuckle. This variety is characterized by flat leaves, strong upright growth, numerous flowering branches, and an extended bud stage, which effectively reduces harvesting costs significantly increasing yield per unit area. Its bud color is consistent with traditional honeysuckle, with excellent internal and external quality, demonstrating excellent internal and external quality, as well as outstanding agronomic traits and processing adaptability [3]. ‘Red Honeysuckle’ is a wild variant of honeysuckle. Featuring purplish branches and leaves with strong cold resistance, remaining evergreen through winter. Its flower buds are red and highly aromatic. The plant exhibits rapid growth, strong adaptability, and excellent tolerance to drought, waterlogging, cold, and poor soil conditions. It represents a superior germplasm resource with medicinal, ornamental, and soil and water conservation value [4]. The former is a high-yield, efficient commercial breeding line, while the latter is a naturally mutated multifunctional variety. Both are representative cultivars of *Lonicera*. Figure 1 displays the appearance and bud morphological characteristics of ‘Beihua No. 1’ and ‘Red Honeysuckle’.

Currently, research on *Lonicera* plants primarily focuses on the flower buds, with relatively few studies concerning the stems, leaves, and branches. Recent studies suggest that the stems, leaves, and flowers of *Lonicera* plants share similarities in their bioactive components, but there are differences mainly in the composition content, which consequently leads to differences in their specific bioactive effects [5]. The young leaves of *Lonicera* are rich in a variety of bioactive compounds, including flavonoids, iridoids, and various organic acids and oils [6,7,8]. As research on *Lonicera* flowers, leaves, and stems advances, their potential applications in fields such as medicine, food, health products, daily chemicals, and feed are increasingly being acknowledged [9,10,11,12]. Studies have shown that leaves of mulberry [13], lotus leaves [14], and goji leaves [15] can be processed into health-promoting tea beverages. However, the bioactive components, volatile profiles, and potential health benefits of tea products made from *L. japonica* tender leaves have yet to be reported. Volatile compounds play a crucial role in shaping the sensory characteristics of tea, but traditional GC-MS technology has limitations such as complex pretreatment and insufficient response to trace flavor substances. GC-IMS technology, which has both high resolution and high sensitivity, can realize rapid and visual fingerprint analysis of volatile components without complex pretreatment, and is especially suitable for the accurate identification of characteristic aromas in complex matrix samples such as tea.

To the best of our knowledge, no study has systematically investigated the quality-related components of *Lonicera* tender bud tea using GC-IMS combined with electronic sensory technology. Additionally, no research has reported the differences between ‘Beihua No. 1’ and ‘Red Honeysuckle’ across different harvesting periods. By combining GC-IMS with the overall sensory evaluation from the electronic nose and tongue, it is possible to complement the analysis and correlate volatile components with sensory quality. We hypothesize that both tea variety and harvesting period significantly affect the volatile compounds, active components, and aroma characteristics of *Lonicera* tender bud tea. Therefore, this study aims to investigate two key aspects: the significant differences in volatile compounds and active components between the two varieties of *Lonicera* tender bud tea, ‘Beihua No. 1’ and ‘Red Honeysuckle’; and how the aroma characteristics and electronic sensory evaluation of *Lonicera* tender bud tea vary across different harvesting periods. To answer these questions, we systematically analyzed the variations in active components and aroma substances across different varieties and harvesting periods using GC-IMS combined with electronic nose and electronic tongue technology. The goal is to provide a theoretical basis for variety selection, harvesting period determination, and the development of novel functional flavored green teas from *Lonicera*.

## 2. Materials and Methods

### 2.1. Chemicals and Reagents

Unless otherwise specified, all reagents were of analytical grade. *n*-alkanones (C_4_–C_9_) were provided by Shanghai Hanon Instruments Co., Ltd. (Shanghai, China). Methanol and acetonitrile, both of chromatographic grade, were obtained from Thermo Fisher Scientific (China) Co., Ltd. (Shanghai, China). Glacial acetic acid (chromatographic grade), vanillin, sodium nitrite, Folin phenol and anthraquinone were provided by Shanghai Macklin Biochemical Technology Co., Ltd. (Shanghai, China). Perchloric acid was sourced from Chengdu Jinshan Reagents Co., Ltd. (Chengdu, China). Phosphoric acid was supplied by Tianjin Fengchuan Chemical Technology Co., Ltd. (Tianjin, China). Concentrated sulfuric acid and sodium carbonate were acquired from Chengdu Kelong Chemical Co., Ltd. (Chengdu, China). Aluminum nitrate was supplied by Shandong Keyuan Biochemical Co., Ltd. (Heze, China). Ninhydrin was obtained from Tianjin Comio Chemical Reagent Co., Ltd. (Tianjin, China). Glucose was provided by Tianjin Baishi Chemical Co., Ltd. (Tianjin, China). Absolute ethanol was acquired from Tianjin Yongda Chemical Reagent Co., Ltd. (Tianjin, China). The reference standards for rutin, gallic acid, luteoloside, oleanolic acid, and chlorogenic acid were sourced from the National Institutes for Food and Drug Control (Beijing, China). Meanwhile, the standards for sweroside and loganin were provided by Anhui Zesheng Technology Co., Ltd. (Anqing, China).

### 2.2. Sample Preparation

**Figure 2:**
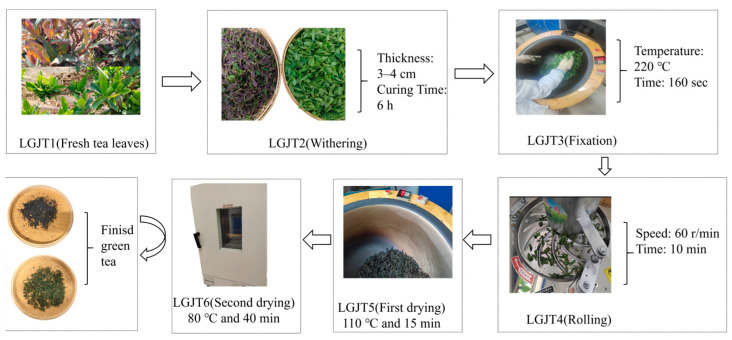
Flowchart of the manufacturing process for *Lonicera japonica* tender bud tea.

The tender buds of the experimental materials, ‘Beihua No.1’ and ‘Red Honeysuckle’, were collected from the honeysuckle experimental field at the Provincial Academy of Agriculture and Forestry Sciences in Xinhua District, Shijiazhuang City, Hebei Province, between April and October 2024. The young shoots, consisting of one bud and two leaves, were harvested and processed into *L. japonica* Green Tea (LJGT). The production procedure involved the following steps: LJGT1 (fresh buds) → LJGT2 (spreading and wilting) → LJGT3 (thermal fixation) → LJGT4 (shaping) → LJGT5 (initial drying) → LJGT6 (final drying). The specific processing conditions for LJGT are presented in Figure 2. The final tea samples were stored at −18 °C for subsequent analysis.

### 2.3. Analysis of Bioactive Compounds

The determination of free amino acids was performed according to the method outlined in the Chinese National Standard GB/T 8314-2013 [16] with appropriate modifications. The soluble protein concentration was determined using the Coomassie Brilliant Blue G-250 dye-binding assay [17]. The content of triterpene saponins was measured based on the method described by Le Bot et al. [18] with slight modifications. Flavonoid levels were assessed through a colorimetric assay [19], while the total tea polyphenol content was quantified with the Folin–Ciocalteu method [20], and soluble sugars were quantified by the anthrone colorimetric method [21]. The contents of luteoloside, chlorogenic acid, loganin, and sweroside, were determined based on the methods specified in the Chinese Pharmacopoeia (2025 Edition, Volume IV) with minor adjustments. luteoloside was extracted using 70% ethanol, while Chlorogenic acid, loganin, and sweroside were extracted by ultrasonication with 50% methanol. The extracts were passed through a 0.22 μm organic filter and analyzed with an HPLC system (LC-16, Shimadzu Corporation, Tokyo, Japan) fitted with a ZPC-C18 column (4.6 mm × 250 mm, 5 μm). The concentrations of the reference standards, along with the corresponding calibration curves and their statistical parameters, are provided in Supplementary Table S1.

### 2.4. Electronic Tongue Measurement

A tea-to-water ratio of 0.5 g per 100 mL was used to prepare the tea infusion, in accordance with the guidelines specified in GB/T 23776-2018. Taste analysis was performed using a taste sensing system (TS-5000Z, Insent Inc., Atsugi, Japan). There were 9 lipid membrane sensors in total, including 6 basic taste sensors (sour, sweet, bitter, salty, umami, and astringent) and 3 aftertaste sensors (bitter aftertaste, umami aftertaste, and astringent aftertaste). In the experiment, the reference buffer (KCl + AgCl, containing tartaric acid) matched with the equipment was used as the cleaning solution and measurement reference, and ultrapure water was used as the blank control to eliminate the background signal. The testing process involved taking 40 mL of tea infusion and placing it in an electronic tongue sample cup. The sample was then analyzed for its main flavor for 120 s, followed by aftertaste assessment for 40 s, and a 10 s cleaning phase. Data collected during the 110—120 s interval were recorded. Each sample was measured 4 times, and three stable measurements were selected for analysis [22].

### 2.5. Electronic Nose Measurement

The aroma characteristics of tea powder were analyzed with a handheld electronic nose system (PEN3, Airsense, Germany) to monitor variations in its odor profile. The device is equipped with ten metal oxide semiconductor sensors, which are sensitive to volatile compounds such as aromatics, nitrogen oxides, sulfur compounds, organic sulfides, and alkanes [23]. Exactly 1 g of tea powder (sieved to 60 mesh) was transferred into a 20 mL headspace vial and hermetically sealed. Before analysis, the vial was incubated at 75 °C for 15 min in a constant-temperature mixer (ZL-100A, Shanghai Zuole Instrument Co., Ltd., Shanghai, China). The syringe temperature was adjusted to 10 °C above the incubation temperature. The operational parameters were set as follows: preparation time of 10 s, injection flow rate of 200 mL/min, sampling duration for analysis of 100 s, and cleaning time of 75 s. The average signal from the stable 80—90 s period was used for data analysis. Each sample was measured three times, and the average value was used for further analysis. All determinations were carried out at ambient temperature.

### 2.6. GC-IMS Analysis

Volatile components in green tea produced from tender buds of two *L. japonica* cultivars across distinct harvest stages were characterized via gas chromatography–ion mobility spectrometry (GC-IMS, FlavourSpec^®^, G.A.S., Dortmund, Germany) fitted with a polar capillary column (30 m × 0.53 mm, film thickness 1 µm). The ion mobility spectrometer drift tube temperature was set at 45 °C, and the injector temperature was 85 °C with a splitless injection mode. The analytical protocol was adapted from a previously documented method [24] with minor adjustments. In brief, 1 g of tea powder was precisely weighed into a 20 mL headspace vial and hermetically sealed. The vial was incubated at 75 °C for 15 min with continuous shaking at 5000 rpm. After incubation, a 500 μL aliquot of headspace gas was drawn into a preheated syringe (70 °C) and introduced into the GC-IMS system. Chromatographic separation was carried out on a polar capillary column maintained at 60 °C, using ultra-purity nitrogen (≥99.999%) as the carrier gas. The flow rate of the carrier gas was programmed in a gradient mode: 2 mL/min within the initial 2 min, 10 mL/min from 2 to 10 min, 50 mL/min over 10–15 min, 100 mL/min during 15–20 min, and 150 mL/min for the last 5 min. High-purity nitrogen was employed as the drift gas at a constant flow rate of 150 mL/min. The detection was performed in positive ion mode.

Retention indices (RI) for volatile compounds were computed with *n*-alkanones (C_4_–C_9_) serving as external calibrants. Identification was based on dual matching of RI and drift time (DT) using a self-built library. Semi-quantification was performed based on peak volumes, represented as the relative percentage of the total peak area. 3D and 2D spectral datasets were analyzed via the Reporter module. The Gallery Plot tool was used to build fingerprint profiles and evaluate VOC pattern differences across samples, and the Dynamic PCA module was adopted to carry out dynamic principal component analysis.

### 2.7. Statistical Analysis

Statistical analysis was performed using one-way analysis of variance (ANOVA) in SPSS software (version 27.0). Differences were considered statistically significant at *p* < 0.05, according to Duncan’s multiple range test. Values with different lowercase letters in figures and tables indicate significant differences between groups. Data visualization, including line charts, bar charts, and radar charts, was conducted using Origin software (version 2021). The proprietary software suite of the aforementioned FlavourSpec® flavor analyzer was employed for spectral processing, compound identification and quantification, and for generating two-dimensional and three-dimensional topographic plots, fingerprint plots, and principal component analysis (PCA) plots. Multivariate statistical analysis of volatile compound data was conducted using the MetaboAnalyst 6.0 online platform, including Principal Component Analysis (PCA), Orthogonal Partial Least Squares Discriminant Analysis (OPLS-DA), and permutation tests, for visualizing sample differences and selecting key feature compounds. Heatmaps and circular clustered heatmaps were constructed using TBtools-II v2.326. software. All experiments were performed in triplicate.

## 3. Results and Discussion

### 3.1. Fundamental Nutritional Compounds Analysis

**Figure 3:**
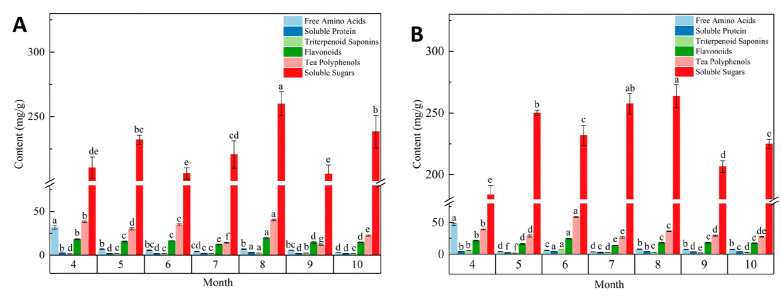
Basic nutritional components in tender bud tea of different *Lonicera japonica* varieties. (**A**) Bar chart of basic nutritional components of ‘Beihua No.1’ Bud Tea from April to October. (**B**) Bar chart of basic nutritional components of ‘Red Honeysuckle’ Bud Tea from April to October.

As illustrated in Figure 3A,B, the basic nutritional components of tender bud tea from the two *Lonicera* cultivars exhibited Distinct dynamic variations over the course of the harvest months. The contents of soluble sugars, tea polyphenols, flavonoids, soluble protein, and triterpenoid saponins all consistent trend of initial increase followed by a subsequent decline. For most of these components, the peaks occurred in August for ‘Beihua No.1’ and in June for ‘Red Honeysuckle’. Free amino acid levels were highest in April (spring) and subsequently declined significantly. This seasonal pattern aligns with previous studies on tea [25,26]. That is, young spring leaves are rich in amino acids, whereas carbon-based metabolites (e.g., soluble sugars, phenolics) accumulate progressively as leaves mature.

The accumulation patterns of nutritional components differed markedly between the two cultivars. This phenological variation may be attributed to distinct genetic backgrounds, such as cultivar-specific differences in the expression timing or activity of key enzymes involved in secondary metabolism (e.g., phenylalanine ammonia-lyase, terpene synthases) [27]. Although free amino acids, triterpenoid saponins, and soluble protein are present in relatively low absolute amounts in tender bud tea, they synergize with tea polyphenols and sugars to modulate the umami, astringency, and mouthfeel of the tea. Studies have shown that an increase in free amino acids (especially theanine) significantly enhances umami and sweetness of tea infusions [28], while phenolic acids are positively correlated with astringency [29].

During the later growth stage, the growth of newly emerging leaves in *Lonicera* tender bud tea slowed and the accumulation of primary metabolites decreased. This may promote the conversion of primary metabolites into secondary metabolites, thereby increasing the levels of compounds such as tea polyphenols and triterpenoid saponins [30]. The above results suggest that the dynamic changes in basic nutritional components in *Lonicera* tender buds across growth stages reveal their intrinsic quality differences.

### 3.2. Pharmaceutically Active Compounds Analysis

**Figure 4:**
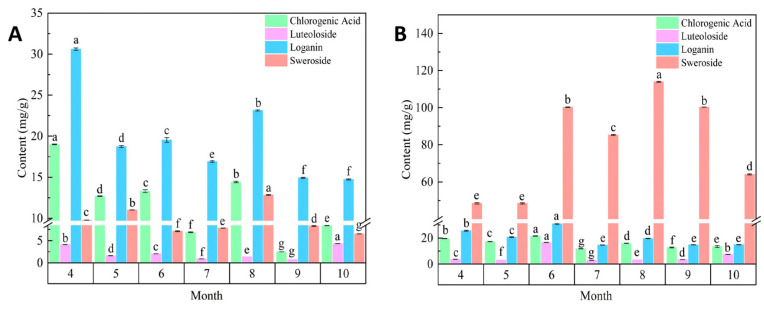
Pharmacologically active components in tender bud tea of different *Lonicera japonica* varieties. (**A**) Bar chart of pharmacologically active components of ‘Beihua No.1’ Bud Tea from April to October. (**B**) Bar chart of pharmacologically active components of ‘Red Honeysuckle’ Bud Tea from April to October.

The health-promoting effects of tea arise from its complex metabolite composition [31]. Tea leaves are rich in natural bioactive components, which impart unique flavor, color, and various health-promoting properties. *Lonicera* tender bud tea is abundant in various characteristic active components and aroma compounds, warranting further research for the development of novel flavored tea products. Chlorogenic acid and luteoloside are the primary functional components used to evaluate the quality of *L. japonica*, and they also act as its key active ingredients, exhibiting antioxidant, anti-inflammatory, and anti-allergic bioactivities. Sweroside and loganin are iridoid compounds detected at relatively high concentrations in the floral, stem, and leaf tissues of *L. japonica*. These compounds exhibit antiviral and blood glucose-regulating activities [32]. As illustrated in Figure 4A,B, the levels of pharmacologically active constituents in *L. japonica* tender bud tea displayed substantial dynamic fluctuations across different harvest months. In the ‘Beihua No.1’ tender bud tea, the contents of chlorogenic acid and loganin showed a general declining trend throughout the growing season. The levels were relatively high in April, followed by a rapid decrease. The changes were more gradual from May to July, with a secondary peak observed in August, after which the contents continued to decline. In contrast, the contents of chlorogenic acid, loganin, and luteoloside in ‘Red Honeysuckle’ followed a pattern of initial increase followed by a decline, peaking in June. The content of sweroside in both cultivars followed a trend of initial increase and subsequent decrease, reaching its highest level in August. However, in August, the concentration in ‘Red Honeysuckle’ was 7.9 times that of ‘Beihua No.1’. The content of luteoloside exhibited different variation patterns between the two cultivars: it peaked in October for ‘Beihua No.1’ and in June for ‘Red Honeysuckle’.

The significant increase in luteoloside content in ‘Beihua No.1’ in October may be attributed to the upregulation of its biosynthetic pathway, which is induced by factors such as biotic stress (e.g., pests and diseases) or abiotic stress (e.g., light variations) [33]. Factors such as cultivation methods [34] and harvesting season [35] influence tea quality, as well as the composition and distribution patterns of metabolites within the leaves themselves [36]. The pharmacologically active components, and consequently the health effects, differ significantly among tender bud teas produced from different cultivars harvested at different times.

**Figure 5:**
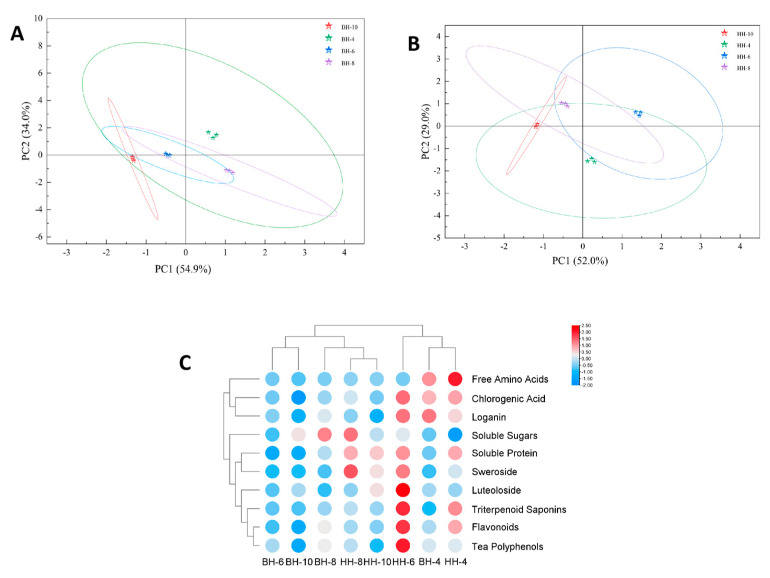
Principal component analysis (PCA) and heatmap of basic nutritional and pharmacologically active components in tender bud tea of different *Lonicera japonica* varieties. (**A**) PCA score plot of monthly variations in ‘Beihua No. 1’; (**B**) PCA score plot of monthly variations in ‘Red Honeysuckle’; and (**C**) heatmap of components in ‘Beihua No. 1’ and ‘Red Honeysuckle’ across April, June, August, and October.

In summary, principal component analysis (PCA; Figure 5A,B) and heatmap analysis (Figure 5C) were performed on the basic nutritional and pharmacologically active components of the tender bud teas from different *L. japonica* cultivars (harvested in April, June, August, and October). As shown in Figure 5A, the April sample of ‘Beihua No.1’ tender bud tea (BH-4) was spatially close to the August sample (BH-8), indicating a similarity in their compositional content, while it was distant from the October sample (BH-10), suggesting a significant difference between them. Concurrently, the June sample (BH-6) was located near the October sample (BH-10), implying that their compositions were relatively similar. For the ‘Beihua No.1’ tender bud tea, PC1 and PC2 accounted for 54.9% and 34.0% of the total variance, respectively, with their combined contribution reaching 88.9%. The results suggest that PCA can effectively differentiate the bioactive components among ‘Beihua No.1’ tender bud teas harvested in different months.

Figure 5B further illustrates that the ‘Red Honeysuckle’ tender bud tea samples from different months show marked compositional discrepancies, suggesting notable fluctuations in their chemical profile over the harvesting period. For this cultivar, PC1 and PC2 explained 52% and 29% of the total variance, respectively, contributing a cumulative total of 81%. These results demonstrate that PCA can effectively distinguish the bioactive components in ‘Red Honeysuckle’ tender bud tea harvested in different months. As shown in the heatmap (Figure 5C), which compares the contents of major bioactive components in the tender bud teas of both ‘Beihua No.1’ and ‘Red Honeysuckle’ across April, June, August, and October, the color intensity intuitively reflects the relative concentration of each component. Overall, the basic nutritional and pharmacologically active components were most prominent in ‘Beihua No.1’ tea harvested in April, while the corresponding components in ‘Red Honeysuckle’ tea reached their peak accumulation in June. This indicates that the accumulation of bioactive components in *L. japonica* tender bud tea exhibits a significant seasonal dependence, and the optimal harvesting period varies between different cultivars.

### 3.3. Electronic Tongue Analysis

**Figure 6:**
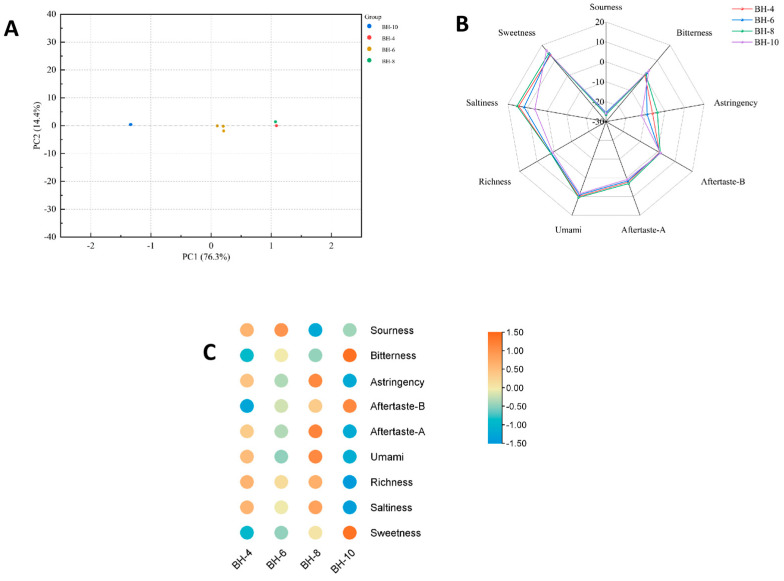
E-tongue data analysis of tender bud tea of ‘Beihua No. 1’. (**A**) PCA score plot; (**B**) radar plot; and (**C**) heatmap.

The taste intensity of *L japonica* tender bud tea varied significantly with the harvesting month (in Supplementary Tables S2 and S3). PCA clearly distinguished samples harvested at different times [37]. As depicted in Figure 6A, the April and August samples formed a tight grouping, suggesting they share similar taste profiles, while they were distant from the October sample, indicating a marked difference in taste—a finding consistent with the PCA results for the active components. According to Figure 6B, the sourness value of the tender bud tea was generally the lowest [38]. The August sample exhibited the highest values in umami, umami aftertaste, astringency, saltiness, and richness. By October, the bitterness value of the tea reached its peak, and its richness was notably lower than in other months. The sweetness value also peaked in October, followed by August. Furthermore, Figure 6C indicates that most taste-related components in the ‘Beihua No.1’ tender bud tea accumulated more abundantly in April and August.

**Figure 7:**
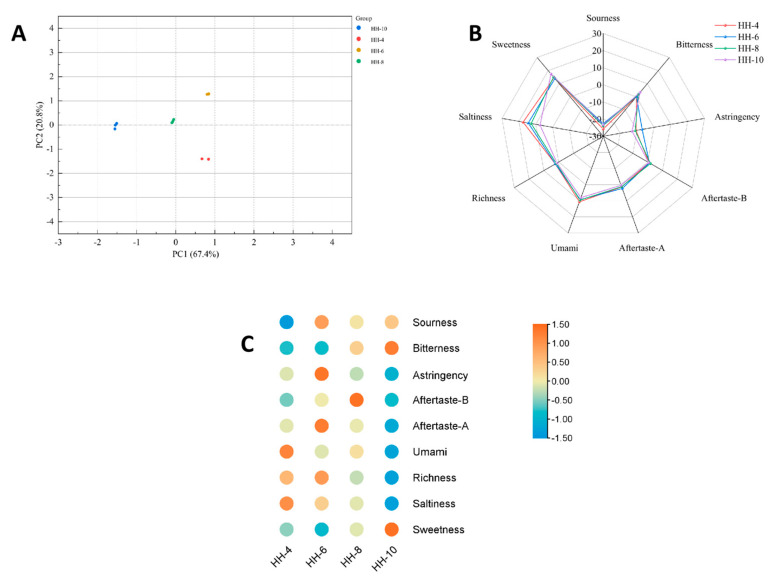
E-tongue data analysis of tender bud tea of ‘Red Honeysuckle’. (**A**) PCA score plot; (**B**) radar plot; and (**C**) heatmap.

Figure 7A shows that the taste components of ‘Red Honeysuckle’ tender bud tea also varied significantly across different harvesting seasons. Among all taste indices, the sourness value was the lowest (Figure 7B), followed by the astringency value. As illustrated in Figure 7C, samples from April and June exhibited relatively high values in umami, umami aftertaste, astringency, saltiness, and richness, indicating a more abundant accumulation of taste components during the early growth stages. In contrast, the later growth stages were primarily characterized by the accumulation of an astringent aftertaste and bitterness. It is noteworthy that tender bud teas of both cultivars exhibited their highest bitterness and sweetness values in October, which may be associated with their higher soluble sugar content and specific functional components [39].

### 3.4. Volatile Compounds Analysis

#### 3.4.1. Electronic Nose Analysis

The electronic nose is a bionic system that mimics biological olfaction, using a sensor array and pattern recognition algorithms to quickly detect and differentiate volatile organic compounds. This technology has found broad application in odor fingerprinting [40,41]. However, while the E-nose provides a holistic response profile of the volatile milieu, it fails to provide detailed compositional or quantitative data. To address this limitation, the present study integrated E-nose analysis with Gas Chromatography–Ion Mobility Spectrometry (GC-IMS) to investigate the fragrance profile and identify specific volatile compounds in *L. japonica* tender bud tea across different harvesting months.

**Figure 8:**
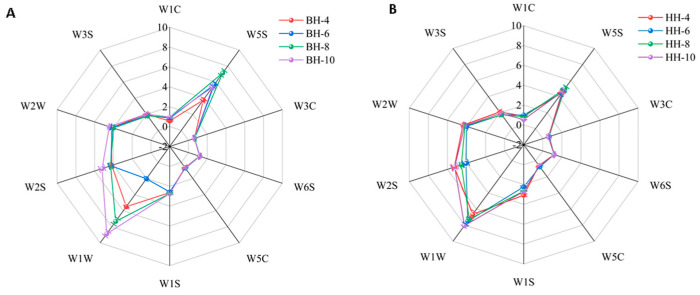
E-nose radar plots of tender bud tea of different *Lonicera japonica* varieties. (**A**) Monthly variations in ‘Beihua No. 1’. (**B**) Monthly variations in ‘Red Honeysuckle’.

As shown in Figure 8, the radar fingerprint plots from the electronic nose revealed distinct response values for *L. japonica* tender bud teas across different harvesting months, indicating variations in their aroma composition profiles. For the ‘Beihua No.1’ tea (Figure 8A), sensors exhibiting notable signal variations included W1W (sensitive to terpenes and organic sulfides), W2S (detecting alcohols and certain aromatic compounds), W2W (responding to aromatic hydrocarbons and organic sulfides), and W5S (sensitive to nitrogen oxides). The April sample (BH-4) showed the lowest response values for most sensors. In the June sample, the response for organic sulfides (W1W) was the lowest, followed by the April and August samples, suggesting a significant influence of harvesting month on organic sulfide content. The August sample exhibited the highest response for the W5S sensor, indicating a relatively higher level of nitrogen oxides during this period. In contrast, the October sample displayed generally higher responses across multiple sensors, implying a gradual accumulation of volatile compounds as the growth period progressed.

The overall variation trend in the volatile components of ‘Red Honeysuckle’ tender bud tea was similar to that observed in ‘Beihua No.1’. As shown in Figure 8B, higher response values from sensors W1W and W2S indicated that organic sulfides, alcohols, and aromatic compounds exhibited notable fluctuations across different months. Sensors W3C (ammonia and amines), W5C (alkenes, aromatic compounds, and polar molecules), and W1C (aromatic hydrocarbons) showed relatively higher responses in the June sample (HH-6), reflecting a more complex aroma profile and a higher content of aromatic substances during this period. The characteristic volatile compounds in the tender bud teas for each month were subsequently identified in greater detail using GC-IMS.

#### 3.4.2. Gas Chromatography–Ion Mobility Spectrometry (GC-IMS) Analysis

##### GC-IMS Analysis of ‘Beihua No. 1’

**Figure 9:**
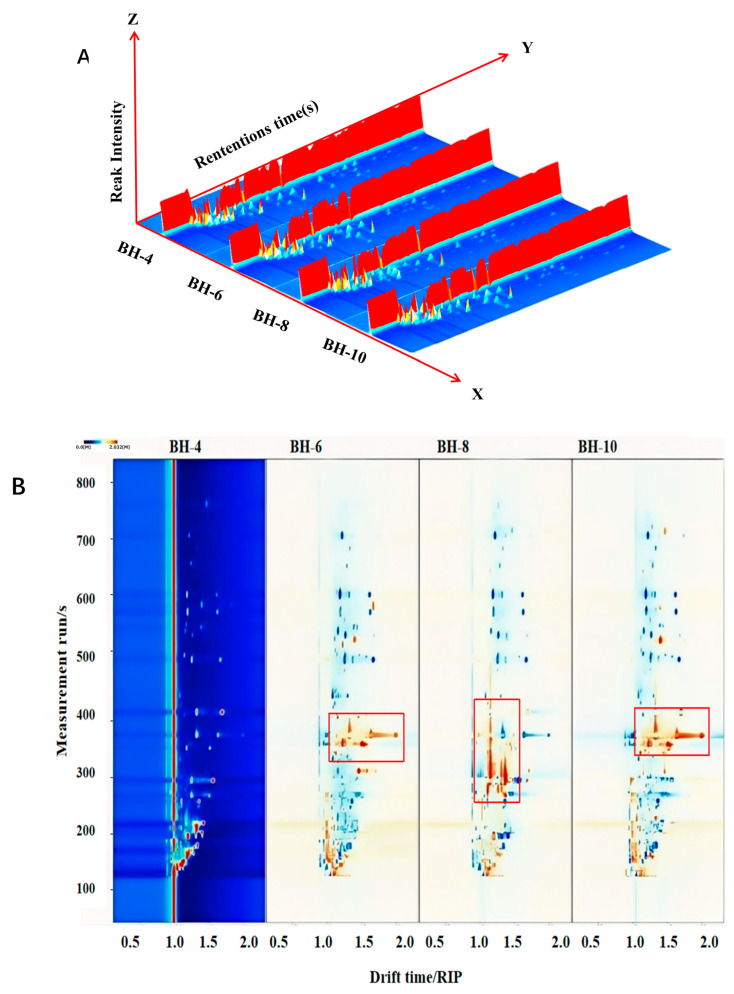

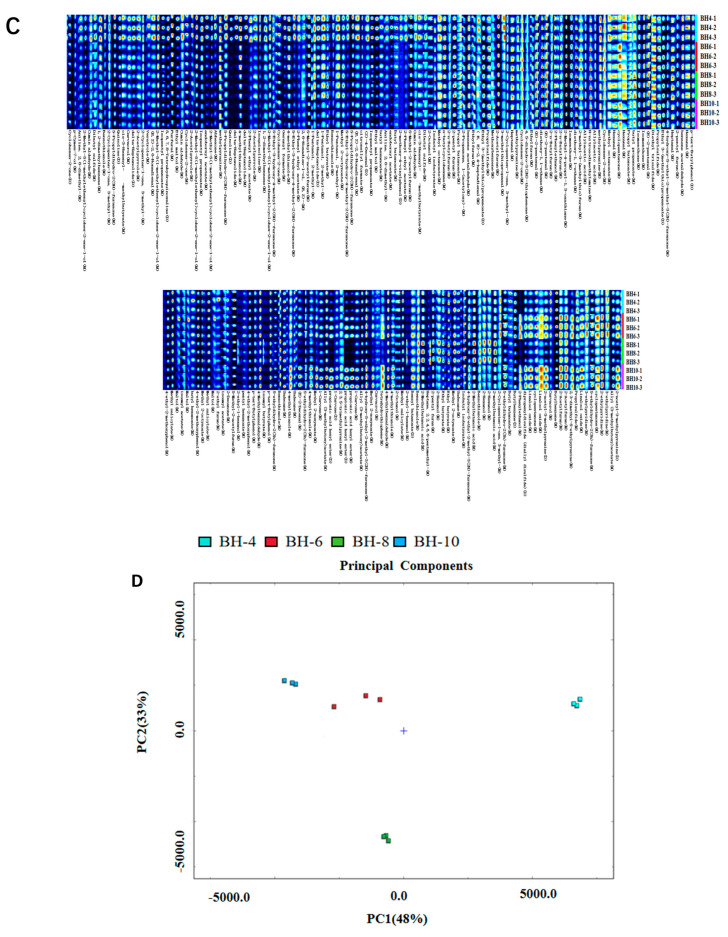
Volatile components analysis of tender bud tea of ‘Beihua No. 1’ across different months. (**A**) 3D topographic map; (**B**) top view of the 3D map; (**C**) fingerprint; and (**D**) principal component analysis (PCA) plot.

GC-IMS was employed to investigate the variations in volatile compounds in the tender buds of ‘Beihua No. 1’ tea over several months, identifying 71 distinct compounds, as detailed in Supplementary Table S4. Among them, esters (28.17%) and heterocyclic compounds (26.76%) were the main categories, together accounting for 54.93% of the total. As shown in Figure 9A,B, the August sample (BH-8) exhibited significant differences from the other groups, while the June (BH-6) and October (BH-10) samples showed similar compositions. This difference was confirmed by subsequent PCA (Figure 9D). The fingerprint map (Figure 9C) further visually revealed distinct variations in volatile profiles across sample categories.

**Figure 10:**
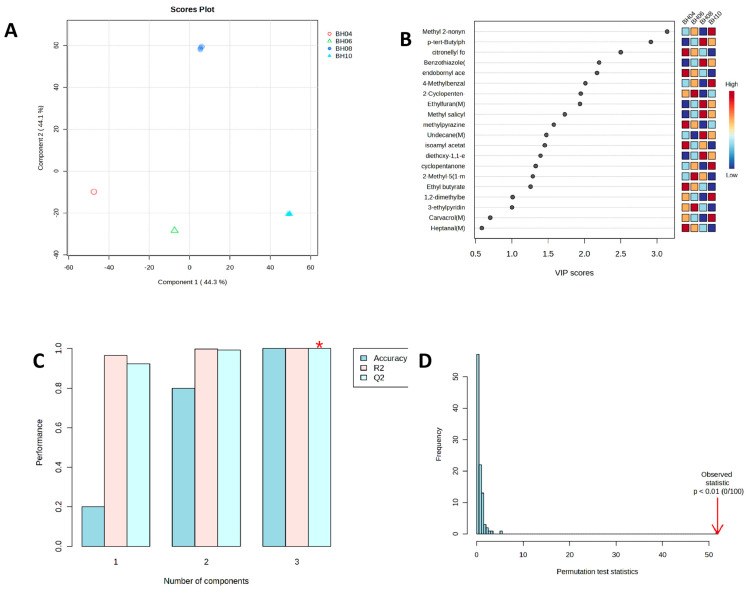
Multivariate statistical analysis of volatile components in tender bud tea of ‘Beihua No.1’. (**A**) Principal component analysis (PCA) score plot; (**B**) variable importance in projection (VIP) plot and corresponding heatmap from OPLS-DA model; (**C**) performance parameters plot of OPLS-DA model, where the asterisk (*) indicates the optimal number of components determined by the model performance metrics (Accuracy, R^2^, and Q^2^); and (**D**) permutation test plot of OPLS-DA model.

To pinpoint key volatile compounds, multivariate statistical analysis was performed using MetaboAnalyst 6.0. PCA revealed that the first two principal components captured 88.4% of the total variance, and the four sample groups were distinctly separated (Figure 10A). The OPLS-DA model identified 18 key compounds with VIP > 1 (such as nonyl prop-2-enoate, 4-methylbenzaldehyde, cyclopentanone, 4-tert-butylphenol, 2-methylpyrazine, and 2-methylfuran), which were associated with floral, fruity, woody, and nutty aromas (Figure 10B). Model evaluation showed that with three principal components, accuracy, R^2^, and Q^2^ were all above 0.99, and Q^2^ was close to R^2^, indicating no overfitting (Figure 10C). Permutation testing (*p* < 0.01, 0/100 permutations) further validated the model’s reliability (Figure 10D). In summary, ‘Beihua No. 1’ is characterized by esters and heterocyclic compounds as the dominant volatile components, with the August sample showing the most volatile profile. A total of 18 volatile compounds with VIP values > 1 were screened out, which can serve as key markers for this variety of *Lonicera* tender bud tea.

##### GC-IMS Analysis of ‘Red Honeysuckle’

**Figure 11:**
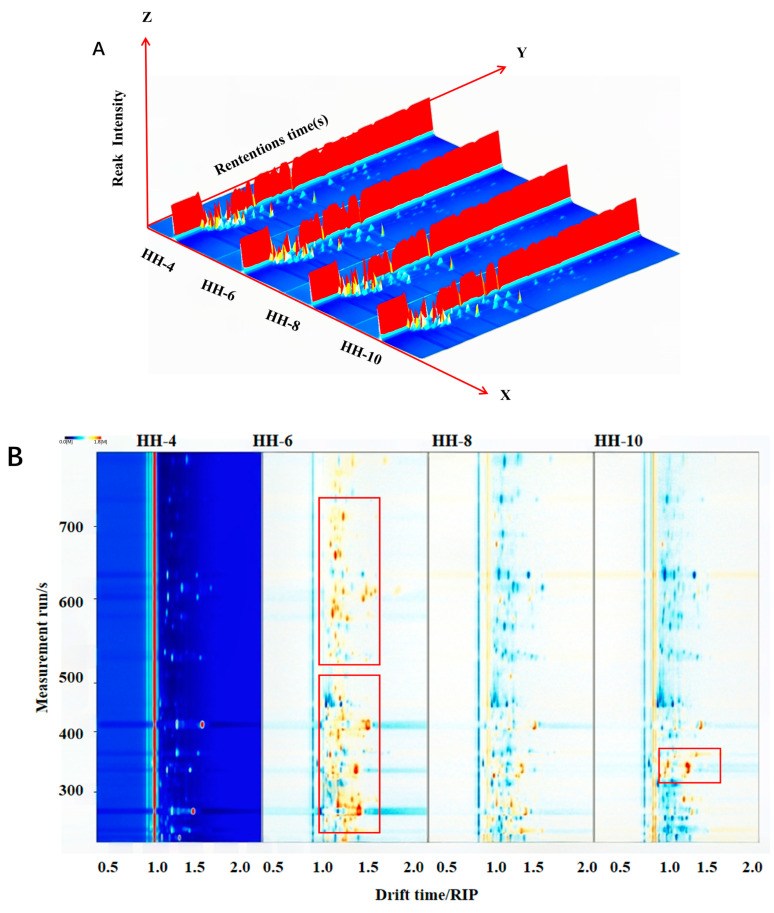

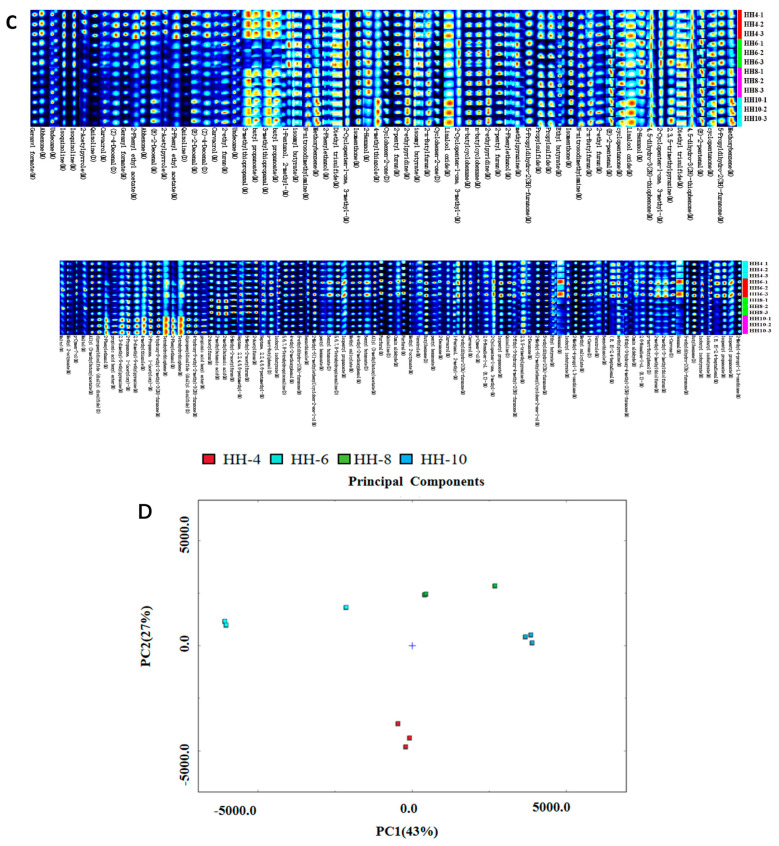
Volatile components analysis of tender bud tea of ‘Red Honeysuckle’ across different months. (**A**) 3D topographic map; (**B**) top view of the 3D map; (**C**) fingerprint; and (**D**) principal component analysis (PCA) plot.

GC-IMS analysis of ‘Red Honeysuckle’ tender bud tea detected 79 volatile constituents (in Supplementary Table S5). Esters (18.92%) and heterocyclic compounds (24.32%) were the major categories, together accounting for 43.24% of the total. The three-dimensional terrain map (Figure 11A) and terrain difference map (Figure 11B) revealed that the June sample (HH-6) deviated notably from the others, which was confirmed by PCA (Figure 11D). The fingerprint map (Figure 11C) further visually revealed distinct variations in volatile profiles across sample categories.

**Figure 12:**
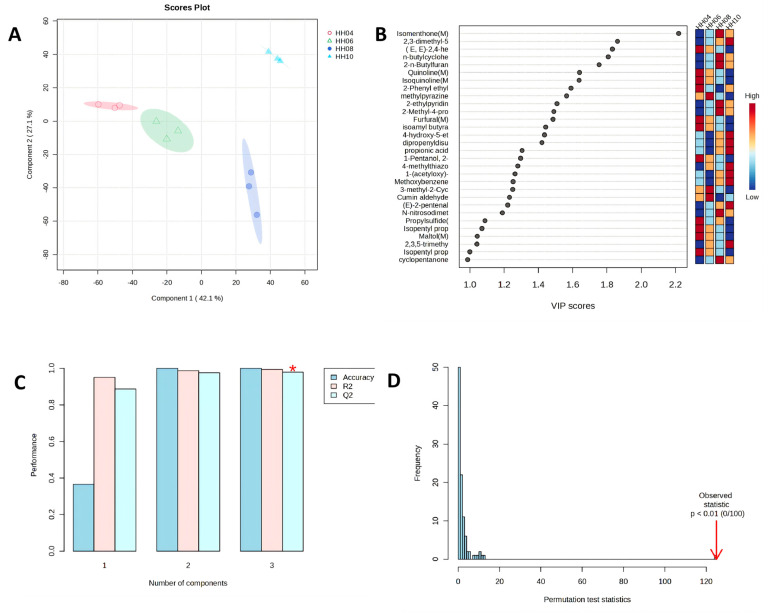
Multivariate statistical analysis of volatile components in tender bud tea of ‘Red Honeysuckle’. (**A**) Principal component analysis (PCA) score plot; (**B**) variable importance in projection (VIP) plot and corresponding heatmap from OPLS-DA model; (**C**) performance parameters plot of OPLS-DA model, where the asterisk (*) indicates the optimal number of components determined by the model performance metrics (Accuracy, R^2^, and Q^2^); and (**D**) permutation test plot of OPLS-DA model.

To identify key volatile compounds, multivariate statistical analysis was performed using MetaboAnalyst 6.0. PCA revealed that the first two principal components captured 69.2% of the total variance, and the four sample groups were distinctly separated (Figure 12A). The OPLS-DA model identified 28 key compounds with VIP > 1, including 2,3,5,6-tetramethylpyrazine, 2-isopropyl-5-methylcyclohexanone, 2-butylfuran, (2E,4E)-hepta-2,4-dienal, and 2-phenylethyl acetate, which were primarily associated with cool minty, nutty roasted, oily, caramel-sweet, and floral-sweet aromas (Figure 12B). Model evaluation showed that with three principal components, accuracy, R^2^, and Q^2^ were all above 0.98, and Q^2^ was close to R^2^, indicating no overfitting (Figure 12C). Permutation testing (*p* < 0.01, 0/100 permutations) further validated the model reliability (Figure 12D). In summary, ‘Red Honeysuckle’ is characterized by esters and heterocyclic compounds as the dominant volatile components, with the June sample showing the most volatile profile. A total of 28 volatile compounds with VIP values > 1 were screened out, which can serve as key markers for this variety of *Lonicera* tender bud tea.

##### Comparative GC-IMS Analysis of ‘Beihua No. 1’ and ‘Red Honeysuckle’

**Figure 13:**
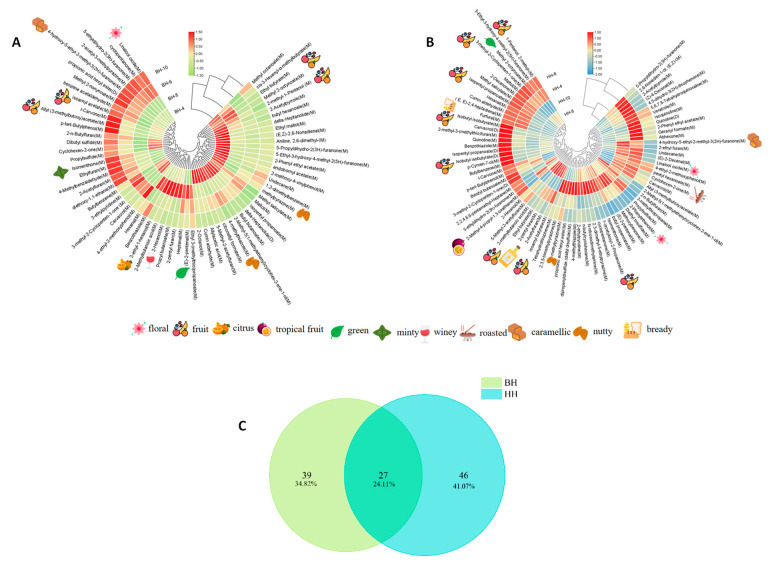
Analysis of volatile components in tender bud tea from two *Lonicera japonica* varieties. (**A**) Circular clustered heatmap for ‘Beihua No. 1’ across different months; (**B**) circular clustered heatmap for ‘Red Honeysuckle’ across different months; and (**C**) Venn diagram of volatile components in both varieties.

The circular clustered heatmaps (Figure 13A,B) further revealed differences in volatile components across months for the two cultivars. For ‘Beihua No.1’ (Figure 13A), the volatile profile transitioned from a dominance of esters and heterocyclics (fruity, floral, caramel-like) in spring to early summer, to an aldehyde-enriched pattern (green/wine-like, fresh) in late summer to autumn. This seasonal variation is consistent with previous reports on tea plants, where spring teas are richer in esters, alcohols, ketones and floral compounds, whereas summer teas contain higher levels of aldehydes and fatty-derived aroma compounds [42,43]. In contrast, for ‘Red Honeysuckle’ (Figure 13B), a more complex volatile matrix was observed, with the June sample showing the highest chemical diversity. Furan-2-carbaldehyde and alkylfurans were identified as characteristic markers, imparting caramel-like, fruity and herbal balsamic notes. These furan compounds have been confirmed as key aroma contributors in various teas: 2-pentylfuran is a key odorant for the chestnut-like aroma of green tea [44]; furan-2-carbaldehyde and 2-methylfuran are positively correlated with chestnut-like intensity in Longjing tea [45]; and furans play an important role in the roasted aroma formation of Wuyi rock tea [46]. Studies on different tea types (green tea, oolong tea) collectively support the general importance of furfural and alkylfurans in constructing complex aroma characteristics.

Venn diagram analysis (Figure 13C) quantified the compositional divergence: 34.82% of the volatiles were unique to ‘Beihua No.1’, 41.07% unique to ‘Red Honeysuckle’, and only 24.11% shared, confirming that the latter possesses a substantially broader volatile repertoire. Overall, the two cultivars exhibited distinctly different flavor architectures. ‘Beihua No.1’ displayed a fresh, fruity and brisk taste profile, aligning with the quality positioning of fresh-style teas. In contrast, ‘Red Honeysuckle’ showed a complex flavor profile characterized by mellow, sweet, caramel-like and nutty notes, making it more suitable for full-bodied tea products that require a sustained sweet aroma character.

## 4. Limitations and Future Perspectives

### 4.1. Limited Cultivar and Sample Size

Basic nutrients of ‘Beihua No.1’ reached the highest level in August, major pharmacological components peaked in April, and loganin content being high. For ‘Red Honeysuckle’, all components were optimal in June, with higher sweroside content. Electronic tongue analysis revealed significant taste differences between the two cultivars. However, only two cultivars, a limited number of picking periods, and a single location and year were examined. Thus, whether these differences can be generalized remains unclear. Future studies should include more cultivars, harvest years, and geographical origins to validate the reproducibility.

### 4.2. Lack of Biological and Clinical Validation

Various active components—including flavonoids, free amino acids, soluble protein, chlorogenic acid, and luteolin—were detected at the chemical level. Nevertheless, no in vitro or in vivo assays were conducted. Any efficacy mentioned herein is cited from existing literature and does not represent a direct conclusion of the present study. Future work should carry out cell-based and animal experiments to verify the functionality.

### 4.3. Single Tea Product Type

Only *Lonicera* tender bud green tea was prepared in this study. ‘Beihua No.1’ contained 71 volatile compounds, whereas ‘Red Honeysuckle’ contained 79, with esters and heterocyclic compounds being dominant. After VIP > 1 screening, 18 key differential components were identified in ‘Beihua No.1’ (e.g., methyl non-2-ynoate, 4-methylbenzaldehyde, cyclopentanone) and 28 in ‘Red Honeysuckle’ (e.g., 2,3,5,6-tetramethylpyrazine, 2-isopropyl-5-methylcyclohexanone, (2E,4E)-hepta-2,4-dienal. These differences in volatile profiles suggest that the two cultivars exhibit distinct flavor characteristics. However, whether *Lonicera* tender leaves are suitable for processing into other tea types (e.g., black tea, oolong tea, dark tea) remains unknown. Future comparative studies across different processing methods are required to determine the most appropriate tea product types to exploit the flavor potential of *Lonicera* tender buds.

## 5. Conclusions

This study systematically analyzed the basic nutritional components, pharmacologically active constituents, and volatile profiles of two *L. japonica* tender bud tea cultivars across different harvesting months, revealing significant varietal and seasonal dynamic differences. First, regarding cultivar differences, ‘Beihua No.1’ was characterized primarily by green, fruity, and nutty flavors, making it suitable for developing fresh and fruity-style tea beverages. In contrast, ‘Red Honeysuckle’ was dominated by caramel, tropical fruit, sweet, and roasted aromas, rendering it more appropriate for crafting mellow and complex aroma tea drinks. Second, in terms of seasonal variation, for ‘Beihua No.1’, spring tender buds contained higher levels of pharmacologically active components (e.g., chlorogenic acid, loganin), indicating potential for functional herbal tea development, while autumn samples were richer in most basic nutritional and volatile compounds, suggesting suitability for teas that balance taste and nutrition. For ‘Red Honeysuckle’, spring tender buds were abundant in basic nutrients, active components, and volatiles, positioning them well for flavored tea beverages with functional attributes and rich aromatic layers; autumn samples exhibited a diverse aroma profile, making them suitable for blended tea drinks with a mellow flavor and pronounced sweetness. In summary, this study not only provides a scientific basis for cultivar selection and harvest time determination of *Lonicera* tender bud tea but also demonstrates the broad applicability of GC-IMS combined with electronic sensory technology in the flavor quality evaluation of herbal teas. More importantly, by revealing the synergistic relationship between aroma compounds and functional components, it opens up a new avenue for the development of healthy teas that balance function and flavor.

## Data Availability

The original contributions presented in this study are included in the article/Supplementary Materials. Further inquiries can be directed to the corresponding authors.

## Supplementary Materials

The following supporting information can be downloaded at: https://www.mdpi.com/article/10.3390/foods15101686/s1, Table S1. HPLC determination of pharmacological active components: concentration of reference standards, linear regression equations, and correlation coefficient. Table S2. E-tongue features of ‘Beihua No.1’. Table S3. E-tongue features of ‘Red Honeysuckle’. Table S4. Identification results of volatile components in ‘Beihua No.1’ tender bud tea. Table S5. Identification results of volatile components in ‘Red Honeysuckle’ tender bud tea.
