# Supplementary material for: Study on Quality Characteristics of Lonicera Tender Bud Tea Based on GC-IMS and Electronic Sensory Technology
Source: Foods. 2026 May 12;15(10):1686. doi: 10.3390/foods15101686 (PMC13205536; doi:10.3390/foods15101686)
Supplement: Supplementary file 1 [file foods-15-01686-s001.zip › Table. S1.pdf]

Table S1

HPLC determination of pharmacological active components: concentration of reference standards, linear regression equations, and correlation coefficient

Substance	Concentration (mg/mL)	Linear Regression Equation	Correlation Coefficient (R^2)
Chlorogenic Acid	0.6	Y = 5E+07x - 10308	1
Loganin	0.52	Y = 3E+07x + 485987	0.9953
Sweroside	0.40	Y = 2E+07x + 93079	0.9987
Luteoloside	0.50	Y = 3E+07x + 299398	0.9985
