# Supplementary material for: Study on Quality Characteristics of Lonicera Tender Bud Tea Based on GC-IMS and Electronic Sensory Technology
Source: Foods. 2026 May 12;15(10):1686. doi: 10.3390/foods15101686 (PMC13205536; doi:10.3390/foods15101686)
Supplement: Supplementary file 1 [file foods-15-01686-s001.zip › Table. S2.pdf]

**Table S2**

E-tongue features of 'Beihua No.1'

| | BH-4 | BH-6 | BH-8 | BH-10 |
|---|---|---|---|---|
| Sourness | $-25.67 \pm 0.36^{a}$ | $-25.50 \pm 0.31^{a}$ | $-25.56 \pm 0.27^{b}$ | $-25.91 \pm 0.27^{ab}$ |
| Bitterness | $0.37 \pm 0.02^{d}$ | $1.56 \pm 0.07^{b}$ | $1.00 \pm 0.06^{c}$ | $3.49 \pm 0.10^{a}$ |
| Astringency | $-6.29 \pm 0.21^{b}$ | $-9.10 \pm 0.19^{c}$ | $-3.81 \pm 0.15^{a}$ | $-11.95 \pm 0.24^{d}$ |
| Aftertaste-B | $1.00 \pm 0.05^{d}$ | $1.29 \pm 0.04^{c}$ | $1.43 \pm 0.03^{b}$ | $1.61 \pm 0.02^{a}$ |
| Aftertaste-A | $2.30 \pm 0.02^{b}$ | $1.68 \pm 0.00^{c}$ | $3.07 \pm 0.04^{a}$ | $0.88 \pm 0.01^{d}$ |
| Umami | $9.99 \pm 0.04^{b}$ | $9.08 \pm 0.03^{c}$ | $10.53 \pm 0.01^{a}$ | $8.47 \pm 0.02^{d}$ |
| Richness | $1.94 \pm 0.02^{a}$ | $1.70 \pm 0.07^{b}$ | $1.97 \pm 0.01^{a}$ | $0.91 \pm 0.02^{c}$ |
| Saltiness | $14.46 \pm 0.08^{b}$ | $11.78 \pm 0.08^{c}$ | $15.56 \pm 0.04^{a}$ | $6.29 \pm 0.07^{d}$ |
| Sweetness | $13.46 \pm 0.12^{d}$ | $14.06 \pm 0.07^{c}$ | $14.72 \pm 0.08^{b}$ | $16.49 \pm 0.07^{a}$ |

Note: Significant different among samples are indicated by different letters ($p < 0.05$).
