# Supplementary material for: Study on Quality Characteristics of Lonicera Tender Bud Tea Based on GC-IMS and Electronic Sensory Technology
Source: Foods. 2026 May 12;15(10):1686. doi: 10.3390/foods15101686 (PMC13205536; doi:10.3390/foods15101686)
Supplement: Supplementary file 1 [file foods-15-01686-s001.zip › Table. S3.pdf]

Table S3

E-tongue features of 'Red Honeysuckle'

	HH-4	HH-6	HH-8	HH-10
Sourness	-25.82 ± 0.12^{d}	-22.66 ± 0.08^{a}	-23.79 ± 0.10^{c}	-23.27 ± 0.19^{b}
Bitterness	0.21 ± 0.02^{c}	0.16 ± 0.01^{d}	1.91 ± 0.03^{b}	3.41 ± 0.03^{a}
Astringency	-10.58 ± 0.09^{b}	-6.94 ± 0.04^{a}	-11.01 ± 0.02^{c}	-12.94 ± 0.09^{d}
Aftertaste-B	0.83 ± 0.03^{c}	1.10 ± 0.07^{b}	1.80 ± 0.02^{a}	0.66 ± 0.01^{d}
Aftertaste-A	1.49 ± 0.09^{b}	2.39 ± 0.02^{a}	1.32 ± 0.01^{c}	0.44 ± 0.01^{d}
Umami	10.64 ± 0.12^{a}	9.37 ± 0.01^{c}	9.62 ± 0.01^{b}	8.12 ± 0.05^{d}
Richness	2.28 ± 0.19^{a}	2.23 ± 0.07^{a}	1.68 ± 0.06^{b}	1.17 ± 0.09^{c}
Saltiness	17.52 ± 0.09^{a}	14.46 ± 0.00^{b}	12.74 ± 0.02^{c}	7.47 ± 0.07^{d}
Sweetness	14.60 ± 0.12^{c}	13.90 ± 0.02^{d}	15.11 ± 0.03^{b}	17.42 ± 0.01^{a}

Note: Significant different among samples are indicated by different letters ($p < 0.05$).
