# Supplementary material for: Study on Quality Characteristics of Lonicera Tender Bud Tea Based on GC-IMS and Electronic Sensory Technology
Source: Foods. 2026 May 12;15(10):1686. doi: 10.3390/foods15101686 (PMC13205536; doi:10.3390/foods15101686)
Supplement: Supplementary file 1 [file foods-15-01686-s001.zip › Table. S4.pdf]

Table S4

Identification results of volatile components in 'Beihua No.1' tender bud tea.

Count	Compound	CAS#	Formula	MW	RI	Rt(sec)	Dt(a.u.)	comment
Esters								
1	endo-bornyl acetate	C125122	$C_{12}H_{20}O_2$	196.3	1294.1	728.899	1.21642	
2	Methyl 2-nonynoate	C111808	$C_{10}H_{16}O_2$	168.2	1297.7	738.558	1.46782	
3	3,7-dimethyloct-6-en-1-yl formate	C105851	$C_{11}H_{20}O_2$	184.3	1285.7	706.683	1.34028	
4	Methyl 2-octynoate	C111126	$C_9H_{14}O_2$	154.2	1221.6	558.062	1.39248	
5	Methyl octanoate	C111115	$C_9H_{18}O_2$	158.2	1130.4	398.715	1.41641	
6	methyl 2-hydroxybenzoate	C119368	$C_8H_8O_3$	152.1	1194.4	504.805	1.14988	
7	ethyl butanoate	C105544	$C_6H_{12}O_2$	116.2	796	131.33	1.20355	
8	3-methylbutyl acetate	C123922	$C_7H_{14}O_2$	130.2	882.7	170.583	1.31396	
9	prop-2-en-1-yl 2-(3-methylbutoxy) acetate	C67634008	$C_{10}H_{18}O_3$	186.3	1242.4	602.363	1.40128	
10	3-methylbutyl propanoate	C105680	$C_8H_{16}O_2$	144.2	980.6	231.58	1.36524	
11	Ethyl 3-(methylthio)propanoate	C13327565	$C_6H_{12}O_2S$	148.2	1130.8	399.257	1.70269	
12	hexyl propanoate (M)	C2445763	$C_9H_{18}O_2$	158.2	1100	356.56	1.99939	Monomer
13	hexyl propanoate (D)	C2445763	$C_9H_{18}O_2$	158.2	1100.8	356.411	2.00223	Dimer
14	Propyl butanoate	C105668	$C_7H_{14}O_2$	130.2	895.4	177.33	1.27129	
15	butyl hexanoate	C626824	$C_{10}H_{20}O_2$	172.3	1194.2	504.357	1.47169	
16	Butyl butanoate	C109217	$C_8H_{16}O_2$	144.2	994.1	241.627	1.81623	
17	2-phenylethyl acetate	C103457	$C_{10}H_{12}O_2$	164.2	1250.5	620.716	1.32734	
18	(Z)-hex-3-en-1-yl 2-methylbutanoate	C53398859	$C_{11}H_{20}O_2$	184.3	1220.9	556.65	1.4358	
19	6-propyloxan-2-one (M)	C3301904	C7H12O2	128.2	1249.9	619.304	1.61684	Monomer
20	6-propyloxan-2-one (D)	C3301904	C7H12O2	128.2	1235.2	586.592	1.62954	Dimer
Heterocyclics								
21	Benzothiazole	C95169	C_7H_5NS	135.2	1223.9	562.76	1.17391	
22	2-ethyl-3-hydroxy-4H-pyran-4-one	C4940118	$C_7H_8O_3$	140.1	1190	496.633	1.66945	
23	3-hydroxy-2-methyl-4H-pyran-4-one	C118718	$C_6H_6O_3$	126.1	1126.3	392.758	1.15173	
24	1-(1H-pyrrol-2-yl) ethanone	C1072839	C_6H_7NO	109.1	1061.4	309.225	1.09036	
25	3-ethylpyridine	C536787	C_7H_9N	107.2	968.6	223.041	1.11626	

26	1-(furan-2-yl) ethanone	C1192627	$C_6H_6O_2$	110.1	902.6	181.348	1.10687
27	2-ethylfuran	C3208160	C_6H_8O	96.1	854.6	156.699	1.04739
28	4-methylthiazole	C693958	C_4H_5NS	99.2	818.1	140.415	1.0537
29	5-ethyl-3-hydroxy-4-methylfuran-2(5H)-one	C698102	$C_7H_{10}O_3$	142.2	1214.4	543.442	1.26633
30	5-propyldihydrofuran-2(3H)-one	C105215	$C_7H_{12}O_2$	128.2	1190.8	498.043	1.25894
31	5-ethyl-4-hydroxy-2-methylfuran-3(2H)-one	C27538096	$C_7H_{10}O_3$	142.2	1100.8	357.495	1.32628
32	2-pentylfuran	C3777693	$C_9H_{14}O$	138.2	994.8	242.129	1.25093
33	1-(3-methylpyrazin-2-yl) ethanone	C23787806	$C_7H_8N_2O$	136.2	1099.5	355.868	1.64172
34	5-ethyldihydrofuran-2(3H)-one	C695067	$C_6H_{10}O_2$	114.1	1080.3	31.462	1.52774
35	1-(5-methylfuran-2-yl) ethanone	C1193799	$C_7H_8O_2$	124.1	994.5	241.878	1.51087
36	2,6-dimethylaniline	C87627	$C_8H_{11}N$	121.2	1192.5	501.176	1.56966
37	4-ethenyl-2-methoxyphenol	C7786610	$C_9H_{10}O_2$	150.2	1294.7	730.617	1.74565
38	2-butylfuran	C4466244	$C_8H_{12}O$	124.2	921	192.148	1.18046
39	2-methylpyrazine	C109080	$C_5H_6N_2$	94.1	840.1	150.014	1.09944
Alcohols							
40	5-isopropyl-2-methylphenol	C499752	$C_{10}H_{14}O$	150.2	1300.9	747.252	1.27927
41	4-ethyl-2-methoxyphenol	C2785899	$C_9H_{12}O_2$	152.2	1275.1	679.637	1.24785
42	2-methyl-5-(prop-1-en-2-yl) cyclohex-2-en-1-ol	C99489	$C_{10}H_{16}O$	152.2	1250.1	619.75	1.1887
43	2-methylpentan-1-ol	C105306	$C_6H_{14}O$	102.2	829.7	145.386	1.29346
44	hexan-2-ol	C626937	$C_6H_{14}O$	102.2	803.7	134.415	1.28557
45	2-ethylhexan-1-ol	C104767	$C_8H_{18}O$	130.2	1027	272.345	1.41111
46	Linalool oxide	C1365191	$C_{10}H_{18}O_2$	170.3	1085.1	337.428	1.26266
47	octan-2-ol	C123966	$C_8H_{18}O$	130.2	997.6	244.39	1.43884
Aldehydes							
48	4-Methylbenzaldehyde	C104870	C_8H_8O	120.2	1080.3	331.462	1.18314
49	Hexanal	C66251	$C_6H_{12}O$	100.2	815.4	139.247	1.25394
50	Heptanal	C111717	$C_7H_{14}O$	114.2	911	186.181	1.33763
51	(E)-2-pentenal	C1576870	C_5H_8O	84.1	755.7	114.532	1.11048
52	(E,Z)-2,6-Nonadienal	C557482	$C_9H_{14}O$	138.2	1190.2	497.087	1.38097
53	phenylacetaldehyde	C122781	C_8H_8O	120.2	1024.2	269.633	1.25736
54	4-isopropylbenzaldehyde	C122032	$C_{10}H_{12}O$	148.2	1234.4	584.977	1.33288
Keto							

55	(2S,5S)-5-methyl-2-propan-2-ylcyclohexan-1-one	C491076	$C_{10}H_{18}O$	154.3	1130.4	398.715	1.33423	
56	(5R)-2-methyl-5-prop-1-en-2-yl cyclohex-2-en-1-one (M)	C2244168	$C_{10}H_{14}O$	150.2	1243.4	599.573	1.81875	Monomer
57	(5R)-2-methyl-5-prop-1-en-2-yl cyclohex-2-en-1-one (D)	C2244168	$C_{10}H_{14}O$	150.2	1241.2	599.768	1.82186	Dimer
58	3-methylcyclopent-2-en-1-one	C2758181	C_6H_8O	96.1	982.3	232.836	1.10687	
59	cyclohex-2-en-1-one (M)	C930687	C_6H_8O	96.1	934.9	200.688	1.10217	Monomer
60	cyclohex-2-en-1-one (D)	C930687	C_6H_8O	96.1	887.9	172.71	1.4028	Dimer
61	cyclopentanone	C120923	C_5H_8O	84.1	803.7	134.415	1.10575	
Aromatics								
62	1,2-dimethylbenzene	C95476	C_8H_{10}	106.2	906.1	183.358	1.06772	
63	Butylbenzene(M)	C104518	$C_{10}H_{14}$	134.2	1025.9	271.26	1.56485	Monomer
64	Butylbenzene(D)	C104518	$C_{10}H_{14}$	134.2	1026.2	271.681	1.5697	Dimer
65	4-tert-butylphenol	C98544	$C_{10}H_{14}O$	150.2	1316.5	791.684	1.46782	
Alkane								
66	Undecane	C1120214	$C_{11}H_{24}$	156.3	1138.1	410.144	1.09997	
Others								
67	1,1-diethoxyethane	C105577	$C_6H_{14}O_2$	118.2	734.8	106.304	1.04265	
68	dipropyl sulfide	C111477	$C_6H_{14}S$	118.2	886.5	172.558	1.16011	
69	2-Methylbutanoic acid	C116530	$C_5H_{10}O_2$	102.1	873.9	166.126	1.20355	
70	Dibutyl sulfide	C544401	$C_8H_{18}S$	146.3	1079.4	330.377	1.30242	
71	pent-4-enoic acid	C591800	$C_5H_8O_2$	100.1	919.7	191.324	1.42596	

Note: MW: Molecular weight; RI: retention index; Rt(set): Retention time; Dt(au): drift time M: monomers; D: dimers.
