# Supplementary material for: Study on Quality Characteristics of Lonicera Tender Bud Tea Based on GC-IMS and Electronic Sensory Technology
Source: Foods. 2026 May 12;15(10):1686. doi: 10.3390/foods15101686 (PMC13205536; doi:10.3390/foods15101686)
Supplement: Supplementary file 1 [file foods-15-01686-s001.zip › Table. S5.pdf]

Table S5

Identification results of volatile components in 'Red Honeysuckle' tender bud tea.

Count	Compound	CAS#	Formula	MW	RI	Rt(sec)	Dt(a.u.)	comment
Esters								
1	(2E)-3,7-dimethylocta-2,6-dien-1-yl formate	C105862	$C_{11}H_{18}O_2$	182.3	1297.8	731.104	1.21746	
2	methyl 2-hydroxybenzoate	C119368	$C_8H_8O_3$	152.1	1196.6	505.566	1.15323	
3	prop-2-en-1-yl 2-(3-methylbutoxy) acetate	C67634008	$C_{10}H_{18}O_3$	186.3	1243.2	599.041	1.39953	
4	Methyl 2-octynoate	C111126	$C_9H_{14}O_2$	154.2	1223.5	557.615	1.39254	
5	hexyl propanoate	C2445763	$C_9H_{18}O_2$	158.2	1100.8	356.56	1.99939	
6	3-methylbutyl butanoate	C106274	$C_9H_{18}O_2$	158.2	1026.7	272.173	1.40382	
7	pentyl hexanoate	C540078	$C_{11}H_{22}O_2$	186.3	1278	680.013	1.53378	
8	2-phenylethyl acetate	C103457	$C_{10}H_{12}O_2$	164.2	1254.5	624.381	1.31842	
9	3-methylbutyl propanoate (M)	C105680	$C_8H_{16}O_2$	144.2	980.6	231.797	1.3638	Monomer
10	3-methylbutyl propanoate (D)	C105680	$C_8H_{16}O_2$	144.2	968.4	222.934	1.36688	Dimer
11	2-methylpropyl 2-methylpropanoa (M)	C97858	$C_8H_{16}O_2$	144.2	912.3	186.497	1.32891	Monomer
12	2-methylpropyl 2-methylpropanoa (D)	C97858	$C_8H_{16}O_2$	144.2	886	171.725	1.31865	Dimer
13	butyl propanoate	C590012	$C_7H_{14}O_2$	130.2	895.7	176.895	1.27862	
14	Ethyl butyrate	C105544	$C_6H_{12}O_2$	116.2	800.1	132.974	1.20592	
15	Benzyl butanoate	C103377	$C_{11}H_{14}O_2$	178.2	1318.8	789.007	1.94207	
16	2-oxopropyl acetate	C592201	$C_5H_8O_3$	116.1	860.8	159.298	1.03729	
Heterocyclics								
17	1-(1H-pyrrol-2-yl) ethanone	C1072839	C_6H_7NO	109.1	1062.7	310.347	1.09468	
18	2-pentylfuran	C3777693	$C_9H_{14}O$	138.2	993.4	241.399	1.254	
19	2-butylfuran	C4466244	$C_8H_{12}O$	124.2	921.3	191.913	1.18113	
20	2-methylpyrazine	C109080	$C_5H_6N_2$	94.1	842.3	150.76	1.10099	
21	5,6,7,8-Tetrahydroquinoxaline	C34413359	$C_8H_{10}N_2$	134.2	1191.9	496.964	1.67117	
22	2-Methyl-4-propyl-1,3-oxathiane	C67715804	$C_8H_{16}OS$	160.3	1131.1	398.241	1.69648	
23	2,3-dimethyl-5-ethylpyrazine	C15707343	$C_8H_{12}N_2$	136.2	1087.6	339.751	1.23075	
24	2,3,5-trimethylpyrazine	C14667551	$C_7H_{10}N_2$	122.2	1005.9	252.343	1.16273	
25	2-methyl-3-(methylthio) furan	C63012975	C_6H_8OS	128.2	940.5	203.977	1.10622	
26	2-ethylpyridine	C100710	C_7H_9N	107.2	903.9	181.573	1.10314	
27	4-methylthiazole	C693958	C_4H_5NS	99.2	818.3	140.368	1.04988	
28	Tetrahydrothiophene	C110010	C_4H_8S	88.2	795.1	131.012	1.04988	

29	2-ethylfuran	C3208160	C_6H_8O	96.1	695.1	91.78	1.04866	
30	5-propyldihydrofuran-2(3H)-one	C105215	$C_7H_{12}O_2$	128.2	1192	497.068	1.25804	
31	5-ethyldihydrofuran-2(3H)-one	C695067	$C_6H_{10}O_2$	114.1	1081.1	331.879	1.18283	
32	5-ethyl-3-hydroxy-4-methylfuran-2(5H)-one	C698102	$C_7H_{10}O_3$	142.2	1233.3	577.832	1.26457	
33	3-hydroxy-2-methyl-4H-pyran-4-one	C118718	$C_6H_6O_3$	126.1	1127	392.342	1.15089	
34	5-ethyl-4-hydroxy-2-methylfuran-3(2H)-one	C27538096	$C_7H_{10}O_3$	142.2	1100.1	355.67	1.32471	
Alcohols								
35	5-isopropyl-2-methylphenol (M)	C499752	$C_{10}H_{14}O$	150.2	1318	786.736	1.27355	Monomer
36	5-isopropyl-2-methylphenol (D)	C499752	$C_{10}H_{14}O$	150.2	1302.9	744.728	1.28477	Dimer
37	4-isopropylbenzyl alcohol	C536607	$C_{10}H_{14}O$	150.2	1287.9	704.991	1.32963	
38	2-methyl-5-(prop-1-en-2-yl) cyclohex-2-en-1-ol	C99489	$C_{10}H_{16}O$	152.2	1237.8	587.357	1.1969	
39	2-Phenylethanol	C60128	$C_8H_{10}O$	122.2	1131.5	398.706	1.49891	
40	(2E,6Z)-nona-2,6-dien-1-ol	C28069729	$C_9H_{16}O$	140.2	1177.2	471.044	1.17943	
41	Linalool oxide	C60047178	$C_{10}H_{18}O_2$	170.3	1087.4	339.539	1.2584	
42	2-methylpentan-1-ol	C105306	$C_6H_{14}O$	102.2	829.7	145.196	1.29126	
43	2-Hexanol	C626937	$C_6H_{14}O$	102.2	803.2	134.181	1.2876	
Aldehydes								
44	(E)-2-pentenal	C1576870	C_5H_8O	84.1	757.7	115.168	1.11936	
45	Furfural	C98011	$C_5H_4O_2$	96.1	837.8	148.761	1.34081	
46	(E)-2-Decenal	C3913813	$C_{10}H_{18}O$	154.3	1300.8	739.051	1.47321	
47	(Z)-dec-4-enal	C21662099	$C_{10}H_{18}O$	154.3	1192	497.068	1.37682	
48	4-isopropylbenzaldehyde	C122032	$C_{10}H_{12}O$	148.2	1238.6	589.185	1.33188	
49	(2E,4E)-hepta-2,4-dienal	C4313035	$C_7H_{10}O$	110.2	1016.9	262.579	1.19189	
50	Hexanal	C66251	$C_6H_{12}O$	100.2	815.8	139.311	1.25225	
51	3-(methylthio) propanal	C3268493	C_4H_8OS	104.2	906	182.804	1.07133	
Keto								
52	cyclopentanone	C120923	C_5H_8O	84.1	803.2	134.181	1.10961	
53	1-(furan-2-yl) ethanone	C1192627	$C_6H_6O_2$	110.1	920.5	191.421	1.42332	
54	cyclohex-2-en-1-one	C930687	C_6H_8O	96.1	887.9	172.71	1.4028	
55	1-(5-methylfuran-2-yl)	C1193799	$C_7H_8O_2$	124.1	995	242.629	1.5126	

	ethanone							
56	(5R)-2-methyl-5-(prop-1-en-2-yl) cyclohex-2-en-1-one	C2244168	$C_{10}H_{14}O$	150.2	1243.4	599.573	1.81875	
57	(2S,5S)-2-isopropyl-5-methyl cyclohexan-1-one	C491076	$C_{10}H_{18}O$	154.3	1131.6	398.939	1.32896	
58	decan-2-one	C693549	$C_{10}H_{20}O$	156.3	1196.5	505.29	1.46899	
59	4,5-dihydrothiophen-3(2H)-one	C1003049	C_4H_6OS	102.2	953.5	212.594	1.18626	
60	3-methylcyclopent-2-en-1-one (M)	C2758181	C_6H_8O	96.1	981.6	232.535	1.10724	Monomer
61	3-methylcyclopent-2-en-1-one (D)	C2758181	C_6H_8O	96.1	969.4	223.672	1.11238	Dimer
62	2-acetyl-4,5-dihydrofuran-3(2H)-one	C698102	$C_7H_{10}O_3$	142.2	1216	542.636	1.26682	
Aromatic hydrocarbon								
63	isoquinoline	C119653	C_9H_7N	129.2	1252.5	619.84	1.19054	
64	Benzothiazole	C95169	C_7H_5NS	135.2	1226.4	563.457	1.17594	
65	4-tert-butylphenol	C98544	$C_{10}H_{14}O$	150.2	1319.5	791.277	1.47096	
66	Quinoline(M)	C91225	C_9H_7N	129.2	1252.5	619.755	1.61787	Monomer
67	Quinoline(D)	C91225	C_9H_7N	129.2	1237.5	586.826	1.62835	Dimer
68	Butylbenzene	C104518	$C_{10}H_{14}$	134.2	1026.2	271.681	1.56186	
69	1,2-dimethoxybenzene	C91167	$C_8H_{10}O_2$	138.2	1194.4	501.547	1.56974	
70	Methoxybenzene	C100663	C_7H_8O	108.1	939.7	203.484	1.04978	
71	4-ethyl-2-methoxyphenol	C2785899	$C_9H_{12}O_2$	152.2	1277.5	678.878	1.24887	
Alkane								
72	Undecane	C1120214	$C_{11}H_{24}$	156.3	1138.7	409.421	1.09041	
73	2,2,4,6,6-pentamethylheptane	C13475826	$C_{12}H_{26}$	170.3	1011.7	257.705	1.09387	
74	butylcyclohexane	C1678939	$C_{10}H_{20}$	140.3	1025.5	270.942	1.25502	
Others								
75	diprop-1-enyl disulfide	C2179579	$C_6H_{10}S_2$	146.3	1099.8	355.204	1.63699	
76	Diethyl trisulfide	C3600246	$C_4H_{10}S_3$	154.3	1131.9	399.404	1.24823	
77	dipropyl sulfide	C111477	$C_6H_{14}S$	118.2	893.5	175.664	1.15753	
78	2-methylbutanoic acid	C116530	$C_5H_{10}O_2$	102.1	877.7	167.54	1.20166	
79	N-nitrosodimethylamine	C62759	$C_2H_6N_2O$	74.1	744	109.585	1.04622	

Note: MW: Molecular weight; RI: retention index; Rt(set): Retention time; Dt(au): Drift time; M: monomers; D: dimers.
